# Effects of a microbial treatment of *Lactobacillus casei*, *Saccharomyces cerevisiae*, and *Lactobacillus plantarum* on blueberry fruit quality, rhizosphere soil properties, and bacterial communities

**DOI:** 10.3389/fmicb.2026.1945124

**Published:** 2026-09-25

**Authors:** Tran Yen Linh Le, Junkyung Lee, Haneul Kim, Hyun-Sik Choi, Sun-Goo Hwang

**Affiliations:** 1 Department of Agricultural Convergence, Sangji University, Wonju-si, Republic of Korea; 2 Department of Applied Plant Science, Sangji University, Wonju-si, Republic of Korea; 3 Department of Scientific Farming Support Team Leader, Hoengseong Agricultural Technology Extension Center, Hoengseong-gun, Gangwon-do, Republic of Korea; 4 Department of Smart Life Science, Sangji University, Wonju-si, Republic of Korea

**Keywords:** 16S rRNA sequencing, blueberry, microbial treatment, soil chemical properties, soil microorganisms

## Abstract

**Introduction:**

Microbial amendments are increasingly used in sustainable crop production, but it remains unclear whether their application is accompanied by coordinated changes in fruit traits, rhizosphere soil conditions, and resident bacterial communities in blueberry cultivation. This study examined whether microbial culture treatment was associated with concurrent changes in blueberry fruit performance, rhizosphere soil chemical properties, and bacterial community characteristics.

**Methods:**

Highbush blueberry (*Vaccinium corymbosum* L. cv. Duke) plants receiving no microbial treatment (NT) were compared with plants receiving a microbial culture treatment (BT) consisting of *Lactobacillus casei*, *Saccharomyces cerevisiae*, and *Lactobacillus plantarum* through drip irrigation. Fruit set date and soluble solids content were evaluated together with harvest-time rhizosphere soil chemical properties, bacterial community characteristics based on full-length 16S rRNA gene sequencing, and exploratory predicted bacterial functional potential.

**Results:**

Relative to NT, BT showed first visible fruit set 10.89 days earlier and higher soluble solids content, increasing from 11.33 ± 1.00 to 15.00 ± 0.71 °Brix. BT was associated with lower electrical conductivity, cation exchange capacity, total nitrogen, ammonium nitrogen, exchangeable K, Ca, Mg, and organic matter, whereas available phosphorus was higher. Soil pH, exchangeable Na, salinity, and nitrate nitrogen did not differ significantly between treatments. Bray–Curtis analysis suggested treatment-associated clustering of bacterial communities; however, PERMANOVA did not detect a statistically significant difference in overall community composition (pseudo-*F* = 1.464, *R*^2^ = 0.268, *p* = 0.100). Within the treatment-responsive bacterial sequence-feature subset, BT showed greater observed richness and diversity, and exploratory taxonomic and predicted MetaCyc pathway analyses identified treatment-associated patterns.

**Discussion:**

Microbial culture treatment was associated with coordinated differences in blueberry fruit traits, rhizosphere nutrient conditions, and bacterial community characteristics. However, the present design does not establish causal relationships among these responses. Because the cultures were applied in growth media without a separate sterile medium control, the effects of viable microorganisms could not be distinguished from potential effects of medium-derived components.

## 1 Introduction

Blueberry (*Vaccinium* spp.) is an economically important fruit crop with increasing commercial value worldwide, and its productivity is closely associated with the physicochemical and biological conditions of the root-zone soil (Che et al., 2022; Li et al., 2020; Zhou et al., 2022). Unlike many other fruit crops, blueberry has a fine, shallow root system and is adapted to acidic, well-drained soils, making it particularly sensitive to variation in soil pH, salinity, nutrient availability, and microbial activity (Lee et al., 2021; Strik and Yarborough, 2005; Zhou et al., 2022). Soil properties including pH, electrical conductivity (EC), organic matter, and nutrient status have been associated with blueberry growth and rhizosphere function (Chen et al., 2019; Pantigoso et al., 2018; Zhou et al., 2022). Therefore, maintaining a favorable rhizosphere environment is an important component of sustainable blueberry production because it supports nutrient availability, microbial community stability, and plant–soil interactions (Che et al., 2022; Schoebitz et al., 2016; Yu et al., 2020).

The rhizosphere bacterial community is closely associated with soil chemical conditions and can vary with plant genotype, cultivation history, and management practices (Che et al., 2022; Lee et al., 2021; Li et al., 2020; Pantigoso et al., 2018). Proteobacteria, Acidobacteria, Actinobacteria, Bacteroidetes, Chloroflexi, and Verrucomicrobia are commonly detected in blueberry-associated soils, although their relative abundances vary among environmental and agronomic conditions (Che et al., 2022; Li et al., 2020; Pantigoso et al., 2018). Among soil properties, pH is widely recognized as an important correlate of bacterial community structure, while EC, cation exchange capacity (CEC), organic matter, nitrogen forms, available phosphorus, and exchangeable cations have also been associated with variation in bacterial community composition (Bahram et al., 2018; Delgado-Baquerizo et al., 2018; Fierer, 2017; Fierer and Jackson, 2006; Lauber et al., 2009; Rousk et al., 2010; Zhou et al., 2022). In blueberry systems, phosphorus and nitrogen availability are particularly relevant because they are associated with plant nutrient status and rhizosphere bacterial composition (George et al., 2018; Nesme and Withers, 2016; Pantigoso et al., 2018; Zhou et al., 2022). Accordingly, joint assessment of soil chemical properties and bacterial communities can provide insight into treatment-associated variation within the blueberry rhizosphere.

Microbial treatments and biofertilizer-based strategies have been investigated as biological approaches for supporting soil quality and sustainable crop production (Adesemoye et al., 2009; Backer et al., 2018; Bhattacharyya and Jha, 2012; Vessey, 2003). Beneficial microorganisms can interact with plant–soil systems through a range of processes related to nutrient availability, organic matter turnover, plant signaling, and interactions with indigenous microbial communities (Compant et al., 2019; Richardson et al., 2009; Vessey, 2003). In blueberry cultivation, applications of beneficial bacterial consortia have been associated with changes in plant growth, fruit quality, and soil nutrient conditions, suggesting potential value for organic or low-input production systems (Yu et al., 2020). Combined applications of microbial consortia and organic or humic substances have also been associated with improved blueberry seedling performance under specific soil conditions (Schoebitz et al., 2016). However, responses to microbial amendments are context-dependent because applied microorganisms interact with resident microbial communities and with the physicochemical characteristics of the soil environment (Backer et al., 2018; Philippot et al., 2013; Vessey, 2003).

Among the soil variables considered in microbial amendment studies, available phosphorus and inorganic nitrogen forms are of particular interest because their concentrations can vary in association with microbial activity, plant nutrient demand, and soil management (George et al., 2018; Kuypers et al., 2018; Nesme and Withers, 2016). However, measurements of available P, ammonium N, and nitrate N alone cannot identify the biological processes responsible for observed treatment-associated differences. Similarly, variation in bacterial community composition may be accompanied by differences in predicted functional potential, but functional inference from 16S rRNA gene data does not directly measure gene expression, enzyme activity, or metabolic flux (Douglas et al., 2020; Langille et al., 2013). Therefore, soil nutrient measurements and predicted bacterial functional profiles should be interpreted as complementary indicators of treatment-associated patterns rather than as direct evidence of specific microbial mechanisms.

Despite growing interest in microbial amendments for blueberry production, it remains unclear whether changes in blueberry fruit traits following microbial culture treatment occur concurrently with measurable differences in rhizosphere soil chemical properties and bacterial community characteristics within the same cultivation system. It is also unclear how harvest-time variation in soil chemical properties covaries with treatment-responsive bacterial groups and their predicted functional potential. Accordingly, the central scientific question of this study was whether microbial culture treatment is associated with coordinated differences in blueberry fruit traits, rhizosphere soil chemical properties, and bacterial community characteristics. We hypothesized that microbial culture treatment would be associated with measurable differences in fruit set and soluble solids content, rhizosphere soil chemical conditions, and bacterial community patterns, together with exploratory differences in predicted bacterial functional potential. To address this question, untreated (NT) and microbial-treated (BT) plants were compared for fruit-related traits, harvest-time rhizosphere soil chemical properties, bacterial community composition based on full-length 16S rRNA gene sequencing, treatment-responsive pre-clustered bacterial sequence features, genus-level abundance patterns, and predicted metabolic pathway profiles. Relationships among soil properties and bacterial community patterns were further evaluated to characterize treatment-associated covariation. Because rhizosphere soil properties and bacterial communities were assessed at harvest, these relationships were interpreted as associations rather than as evidence of causal pathways linking rhizosphere changes to fruit responses.

## 2 Materials and methods

### 2.1 Blueberry cultivation and microbial treatment

The experiment was conducted from 15 February to 25 June 2025 in an unheated plastic greenhouse at a commercial blueberry farm located at 37.4556° N, 128.0700° E in Hoengseong County, Gangwon State, Republic of Korea. Northern highbush blueberry (*Vaccinium corymbosum* L. cv. Duke) was used in this study. The blueberry plants were maintained under the grower’s standard cultivation practices throughout the experimental period. The mean air temperature and relative humidity inside the plastic greenhouse during the cultivation period were 18 °C and 60%, respectively. The experiment consisted of an untreated control (NT) and a microbial treatment (BT). Multiple blueberry plants were maintained under each treatment condition under the same routine cultivation and irrigation practices, except for the microbial treatment applied to BT. For fruit set date and soluble solids content, nine individual plants per treatment were evaluated independently (*n* = 9 per treatment). For rhizosphere soil chemical and bacterial community analyses, three additional individual plants per treatment were sampled independently (*n* = 3 per treatment); these plants were distinct from those used for fruit measurements. Each individual plant represented one biological replicate for the corresponding analysis, and no pooling among plants was performed. Detailed records of the exact spatial arrangement and randomization of individual plants within the commercial greenhouse were not available for retrospective verification; therefore, no unverified randomization scheme or spatial layout was assumed.

The microbial treatment consisted of a mixed culture of *Lactobacillus casei* and *Saccharomyces cerevisiae*, together with a separately prepared *Lactobacillus plantarum* culture. *L. casei* and *L. plantarum* were propagated in de Man, Rogosa and Sharpe (MRS) broth (BD Difco, Sparks, MD, USA), whereas *S. cerevisiae* was cultured in yeast extract–peptone–dextrose (YPD) broth containing 10 g L^−1^ yeast extract, 20 g L^−1^ peptone, and 20 g L^−1^ glucose. The lactic acid bacteria were incubated at 30 °C for 24–48 h, while *S. cerevisiae* was cultured at 30 °C for 24 h with shaking. Viable cell concentrations were determined by serial dilution and plate counting using MRS agar for Lactobacillus spp. and YPD agar for *S. cerevisiae*. After cultivation, the *L. casei* and *S. cerevisiae* cultures were combined to prepare a mixed culture with a total viable cell concentration of 1 × 10^8^ CFU mL^−1^. In contrast, the *L. plantarum* inoculum was maintained as a separate culture and independently adjusted to 1 × 10^9^ CFU mL^−1^. The separate preparation of *L. plantarum* allowed its higher target viable-cell concentration to be controlled independently while maintaining the intended composition of the *L. casei*–*S. cerevisiae* mixed culture prior to application.

During the treatment period, the microbial cultures were delivered to the BT treatment through the drip-irrigation system in repeated divided applications. The cumulative application volumes recorded over the treatment period were 514 L for the *L. casei*–*S. cerevisiae* mixed culture and 156 L for the separately prepared *L. plantarum* culture. These values represent the total volumes of inoculum applied over the treatment period and were not treated as fixed volumes per individual irrigation event. The NT treatment was maintained under the same routine irrigation conditions but received neither the microbial cultures nor the corresponding culture media. No separate sterile culture-medium control was included in the experimental design.

At the end of the cultivation period, rhizosphere soil was collected from the root zones of the blueberry plants selected for soil and bacterial community analyses. Loosely attached soil was removed by gentle shaking, and soil tightly adhering to the roots was collected as rhizosphere soil. The samples were transported to the laboratory under cooled conditions. Subsamples designated for bacterial community analysis were stored at −80 °C, whereas samples used for soil chemical analyses were processed according to the procedures described in the corresponding section below.

### 2.2 Fruit set date and soluble solids content

Nine representative blueberry plants were selected from each treatment for the evaluation of fruit set date and soluble solids content (SSC). Each selected plant was treated as an independent biological replicate for these fruit-related measurements, resulting in nine biological replicates per treatment (NT, *n* = 9; BT, *n* = 9). Measurements were obtained individually from each plant, and individual plant values were used directly for statistical analysis without pooling or averaging among plants.

Fruit set date was visually determined for each selected plant and recorded as the number of days after treatment initiation (DAT) to the first visible formation of developing berries following flowering.

At harvest, representative fully ripe berries were collected separately from each selected plant. The berries were gently homogenized, and juice was extracted from the berry pulp. Soluble solids content (SSC) was measured from freshly extracted juice using a portable digital refractometer (PAL-1, ATAGO Co., Ltd., Tokyo, Japan) equipped with automatic temperature compensation. The refractometer was calibrated with distilled water before measurement, and SSC was expressed as °Brix. The refractometric determination of soluble solids was performed according to established methods for fruit and vegetable products (Li et al., 2024; Wang et al., 2023).

Fruit set date and SSC data are presented as the mean ± standard deviation of nine individual biological replicates per treatment. Differences between the untreated control (NT) and microbial treatment (BT) were evaluated using two-sided Welch’s two-sample *t*-tests based on the individual plant values (*n* = 9 per treatment). Statistical significance was determined at *p* < 0.05.

### 2.3 Soil chemical analysis

At the end of the cultivation period, rhizosphere soil samples were collected independently from three representative blueberry plants in each treatment. These plants were distinct from those used for fruit set date and soluble solids content measurements. Rhizosphere soil obtained from each individual plant was treated as one biological replicate, resulting in three biological replicates per treatment (NT, *n* = 3; BT, *n* = 3) and a total of six soil samples. Soil collected from different plants was processed separately and was not pooled.

Loosely attached soil was removed from the roots by gentle shaking, whereas soil remaining closely associated with the root surface was collected as rhizosphere soil. Each individual rhizosphere soil sample was thoroughly homogenized separately after collection. Subsamples used for ammonium nitrogen (NH₄^+^–N) and nitrate nitrogen (NO₃^−^–N) analyses were maintained fresh, whereas the remaining soil was air-dried, gently crushed, and passed through a 2-mm sieve prior to chemical analysis.

Soil chemical properties were analyzed according to the procedures described by Lee et al. (2023), with minor modifications. Soil pH and salinity were determined using a 1:5 (w/v) soil-to-distilled-water suspension, whereas electrical conductivity (EC) was determined using a 1:1 (w/v) soil-to-distilled-water suspension. Total nitrogen (T-N) was determined using the Kjeldahl method and reported as percentage (%), and available phosphorus (A-P) was measured using the Lancaster extraction method and reported as mg kg^−1^. Exchangeable K, Ca, Mg, and Na were extracted with 1 M ammonium acetate (NH₄OAc; pH 7.0) and quantified by inductively coupled plasma optical emission spectrometry (ICP-OES), with concentrations reported as cmolc kg^−1^. Cation-exchange capacity (CEC) was determined using the ammonium acetate saturation method and reported as cmolc kg^−1^, whereas organic matter (OM) was determined using the Tyurin dichromate oxidation method and reported as percentage (%) (Lee et al., 2023).

For inorganic nitrogen analysis, NH₄^+^–N and NO₃^−^–N were extracted from fresh soil using 2 M KCl at a soil-to-extractant ratio of 1:5 (w/v). The extracts were filtered and analyzed colorimetrically, with NH₄^+^–N determined using the indophenol blue method and NO₃^−^–N determined after cadmium reduction (Keeney and Nelson, 1982). Soil chemical data are presented as the mean ± standard deviation of three individual-plant biological replicates per treatment (NT, *n* = 3; BT, *n* = 3). Differences between NT and BT for each soil chemical property were evaluated using one-way analysis of variance (ANOVA), with treatment as the single factor. Each analysis included six sample-level observations, corresponding to 1 degree of freedom for treatment and 4 residual degrees of freedom. Statistical significance was determined at *p* < 0.05.

### 2.4 Soil DNA extraction and full-length 16S rRNA sequencing

Total community DNA was extracted separately from each of the six individual-plant rhizosphere soil samples described above (NT, *n* = 3; BT, *n* = 3). Each soil sample was derived from a single blueberry plant and represented one biological replicate. For each sample, DNA was extracted from 0.25 g of homogenized blueberry rhizosphere soil using the DNeasy PowerSoil Pro Kit (QIAGEN, Hilden, Germany) according to the manufacturer’s instructions. Soil samples were subjected to mechanical and chemical lysis in PowerBead Pro tubes, followed by inhibitor removal and silica membrane-based DNA purification. Purified DNA was eluted in nuclease-free water and stored at −20 °C until further analysis.

DNA concentration was determined using a Qubit 4 Fluorometer with the Qubit dsDNA High Sensitivity Assay Kit (Thermo Fisher Scientific, Waltham, MA, USA), and DNA purity was assessed using a NanoDrop spectrophotometer (Thermo Fisher Scientific). DNA integrity was evaluated by agarose gel electrophoresis. Only DNA samples with sufficient concentration, acceptable purity, and no apparent degradation were used for subsequent full-length 16S rRNA gene amplification.

The full-length bacterial 16S rRNA gene spanning the V1–V9 regions was amplified using barcoded universal primers corresponding to the 27F and 1492R positions. The forward primer sequence was 5′-AGRGTTYGATYMTGGCTCAG-3′, and the reverse primer sequence was 5′-RGYTACCTTGTTACGACTT-3′. Sample-specific barcode sequences and PacBio-compatible adapter sequences were incorporated at the 5′ ends of the primers to enable multiplex sequencing.

PCR amplification was performed using 2 ng of genomic DNA in a 50-μL reaction containing 10 × LA PCR Buffer II (Mg^2+^-free), 2.5 mM dNTP mix, 2.5 mM MgCl₂, 500 nM each of the forward and reverse primers, and 5 U of TaKaRa LA Taq (Takara Bio, Kusatsu, Japan). Thermal cycling consisted of an initial denaturation at 94 °C for 5 min, followed by 25 cycles of denaturation at 94 °C for 30 s, annealing at 53 °C for 30 s, and extension at 72 °C for 90 s, with a final extension at 72 °C for 5 min. Each biological replicate was amplified independently in a single barcoded PCR reaction, and no technical PCR replicates were pooled within samples.

Following amplification, the PCR amplicon from each of the six biological replicates was quantified separately and normalized to an equivalent molar concentration. The normalized, individually barcoded amplicons were subsequently pooled for library preparation and purified using SMRTbell Cleanup Beads (Pacific Biosciences of California, Inc., Menlo Park, CA, USA) according to the manufacturer’s protocol. The sequencing-ready library was sequenced on a PacBio Sequel IIe system (Pacific Biosciences of California, Inc.) using single-molecule real-time (SMRT) sequencing at Macrogen (Seoul, Republic of Korea). Circular consensus sequencing was used to generate high-fidelity (HiFi) reads. The resulting reads were demultiplexed according to their sample-specific barcodes, and primer and adapter sequences were removed before downstream bacterial community analysis.

### 2.5 Processing of 16S rRNA gene sequencing data

Raw PacBio HiFi reads generated from full-length bacterial 16S rRNA gene amplicons were processed using an in-house bioinformatics workflow based on fastp and mothur version 1.48.0 (Chen et al., 2018; Schloss et al., 2009). Each rhizosphere soil sample derived from an individual blueberry plant was treated as one biological replicate and one sample-level analytical unit for bacterial community analysis. Three individual biological replicates were analyzed per treatment (NT, *n* = 3; BT, *n* = 3), resulting in six independent sample-level analytical units.

Because the PacBio HiFi dataset consisted of single-end long reads, paired-end read assembly was not required. Raw HiFi reads were initially subjected to quality filtering using fastp. Reads were filtered using a minimum Phred quality score of 20, and reads containing more than 30% of bases with Phred scores below 20 were discarded. Right-end quality trimming was performed using a sliding window of four bases with a mean quality threshold of 20. Adapter trimming was disabled during this step. Reads shorter than 1,200 bp after quality filtering were removed.

Quality-filtered reads were converted from FASTQ to FASTA format, and sample-specific group and count files were generated to preserve the sample origin and abundance information of each sequence. The sequences were dereplicated using the unique.seqs command in mothur, and the resulting representative sequences were aligned against the SILVA v138 16S rRNA gene reference alignment (Quast et al., 2012). Sequences aligning in both forward and reverse orientations were retained for downstream processing. All retained sequences were subsequently subjected to the same downstream quality-control procedures.

Alignment summaries were generated using the summary.seqs command, and conservative alignment boundaries were determined from the distribution of aligned sequence start and end positions. Specifically, the first quartile of start positions and the third quartile of end positions were used as alignment boundaries for sequence screening. Aligned sequences were screened using the screen.seqs command. Sequences were retained only when they satisfied the defined alignment boundaries, contained no ambiguous bases, had homopolymer stretches of ≤8 bp, and had lengths between 1,200 and 1,800 bp. Alignment columns containing only gap characters were subsequently removed using the filter.seqs command, after which the filtered sequences were dereplicated again.

To reduce residual sequencing errors while preserving biologically meaningful sequence variation, the dereplicated sequences were pre-clustered using the pre.cluster command with a maximum difference threshold of two nucleotides (diffs = 2) (Huse et al., 2010). Accordingly, the sequence-level units retained for downstream community analyses were defined as quality-controlled, pre-clustered bacterial sequence features, rather than raw unique sequences or conventional operational taxonomic units. Final sample-by-feature abundance tables were generated from the quality-controlled, pre-clustered, non-chimeric bacterial sequences and were used for subsequent ecological analyses.

Putative chimeric sequences were detected and removed using the VSEARCH algorithm implemented in mothur through the chimera.vsearch command (Rognes et al., 2016). Chimera detection was performed with the dereplicate = T option to account for sample-specific sequence abundances during chimera screening. Taxonomic classification of the resulting non-chimeric sequences was performed in mothur using the Ribosomal Database Project (RDP) v18 reference database (Cole et al., 2014; Wang et al., 2007), with a bootstrap confidence threshold of 80%. Sequences assigned to chloroplasts, mitochondria, Archaea, Eukaryota, or unclassified/non-target lineages were removed using the remove.lineage command. The remaining bacterial sequences constituted the final quality-controlled bacterial dataset.

Final count and sample-by-feature abundance tables were generated from the quality-controlled, pre-clustered, non-chimeric bacterial sequences for downstream ecological analyses. Read retention throughout the preprocessing workflow was evaluated using fastp quality-control reports and mothur sequence-summary outputs. The resulting sample-by-feature abundance table and corresponding taxonomic assignments were used for subsequent analyses of bacterial community composition, diversity, treatment-associated sequence features, and relationships with rhizosphere soil chemical properties.

### 2.6 Associations between soil properties and bacterial community composition

The relationships between rhizosphere soil chemical properties and bacterial community composition were evaluated in R using the vegan and phyloseq packages (Mantel, 1967; McMurdie and Holmes, 2013; Oksanen et al., 2001). The final bacterial sequence-feature abundance matrix obtained from the reprocessed dataset, the corresponding taxonomic annotation table, and the soil chemical dataset were matched according to shared sample identifiers before analysis. The bacterial sequence features used in these analyses corresponded to the quality-controlled, pre-clustered, non-chimeric bacterial sequences described above. Each sample represented rhizosphere soil collected from one individual blueberry plant, and three independent biological replicates were analyzed per treatment (NT, *n* = 3; BT, *n* = 3).

For Mantel analyses, bacterial sequence-feature abundances were converted to relative abundances within each sample to account for differences in total sequence abundance among samples. No additional mathematical transformation was applied. Euclidean distance matrices were calculated from the resulting relative-abundance profiles to quantify pairwise differences in proportional bacterial abundance among samples. For each soil chemical property, a corresponding Euclidean distance matrix was calculated separately. Associations between bacterial community composition and individual soil properties were then evaluated using Mantel tests based on Spearman rank correlations with 9,999 permutations. Bray–Curtis dissimilarity was analyzed separately to evaluate overall bacterial community dissimilarity between samples. Differences in overall bacterial community composition between NT and BT were formally evaluated by permutational multivariate analysis of variance (PERMANOVA) based on Bray–Curtis dissimilarities using the adonis2 function in the vegan package with 999 permutations. The analysis was based on six individual-plant rhizosphere samples (NT, *n* = 3; BT, *n* = 3), corresponding to 1 treatment degree of freedom and 4 residual degrees of freedom. Homogeneity of multivariate dispersion was additionally assessed using the betadisper function followed by a permutation test.

Phylum-level Mantel tests were additionally used as an exploratory screening step to identify bacterial groups potentially associated with individual soil chemical properties and to reduce the number of taxonomic groups considered in the subsequent constrained ordination. Phyla represented by fewer than two bacterial sequence features were excluded. Taxonomic groups showing nominal Mantel *p* ≤ 0.05 were retained for subsequent exploratory CCA. No multiple-testing adjustment was applied at this screening stage; therefore, these Mantel associations were interpreted as nominal exploratory associations rather than confirmatory statistical evidence.

Canonical correspondence analysis (CCA) was subsequently performed to examine multivariate covariation between the Mantel-selected bacterial groups and soil chemical properties (Ter Braak, 1986). Environmental variables with zero variance were removed before analysis, and multicollinearity among the remaining variables was evaluated using variance inflation factors (VIFs). Variables with VIF values > 10 were sequentially excluded to reduce redundancy among environmental predictors. The overall CCA model, canonical axes, and environmental terms were independently evaluated using permutation tests with 999 permutations. Environmental vectors were additionally fitted to the ordination using the envfit function with 999 permutations. CCA biplots were generated using sample scores, selected bacterial-group scores, and fitted soil environmental vectors. Given the limited number of biological replicates, the Mantel and CCA analyses were interpreted as exploratory assessments of covariation between soil chemical properties and bacterial community composition rather than as evidence of causal relationships.

Bacterial groups identified through the exploratory analyses were further examined at the phylum and genus levels using phyloseq (McMurdie and Holmes, 2013). Sequence features were agglomerated according to their taxonomic assignments, and relative abundances were calculated within each sample. For phyla of interest, the corresponding sequence features were further summarized at the genus level to examine treatment-associated compositional patterns. For genus-level exploratory analysis, the relative abundance of each genus or unclassified taxonomic group across the six biological samples was evaluated in relation to treatment group using Spearman rank correlation. Taxa with a nominal *p* < 0.05 were retained for subsequent descriptive comparison. Positive Spearman correlation coefficients indicated higher relative abundance in BT, whereas negative coefficients indicated higher relative abundance in NT. No multiple-testing correction was applied to these genus-level comparisons; therefore, the identified taxa were interpreted as exploratory treatment-associated abundance patterns rather than as statistically confirmed differentially abundant taxa. Genus-level composition was visualized using treatment-wise relative-abundance summaries and mean ± standard deviation plots.

### 2.7 Prediction and analysis of bacterial functional potential

The potential functional profiles associated with the selected bacterial sequence-feature dataset were predicted using PICRUSt2 version 2.5.1 (Douglas et al., 2020). The final quality-controlled, pre-clustered, non-chimeric representative 16S rRNA gene sequences were converted into PICRUSt2-compatible FASTA format, and sequence identifiers were matched to the corresponding feature abundance table. An SRS-normalized abundance table containing the union of selected bacterial sequence features was converted from comma-separated to tab-delimited format and used as the input abundance table for functional prediction (Beule and Karlovsky, 2020).

PICRUSt2 was run using the picrust2_pipeline.py workflow with 40 processing threads. Representative sequences were placed into the PICRUSt2 reference phylogeny, gene-family copy numbers were predicted, and predicted functional profiles associated with the selected bacterial sequence-feature set were subsequently generated. Unstratified predicted abundance tables were obtained for MetaCyc pathways, and functional descriptions were added using the add_descriptions.py utility provided with PICRUSt2.

Pairwise comparisons of predicted MetaCyc pathway abundances between untreated (NT) and microbial-treated (BT) rhizosphere soil samples were performed using a custom R script. Each rhizosphere soil sample represented one individual-plant biological replicate, with three biological replicates analyzed per treatment (NT, *n* = 3; BT, *n* = 3). Differences in predicted pathway abundance between treatments were evaluated using two-sample t-tests at a nominal significance threshold of *p* < 0.05. No multiple-testing correction was applied to these pathway-level comparisons; therefore, nominal *p* values were used for exploratory screening and were not interpreted as confirmatory evidence of statistically significant functional differences between treatments. For graphical summary, up to 50 pathways meeting the nominal *p* < 0.05 criterion were retained and ranked according to total predicted abundance.

Predicted functional profiles were interpreted as estimates of the functional potential associated with the selected bacterial sequence-feature set inferred from 16S rRNA gene sequence information, rather than as direct measurements of gene abundance, gene expression, enzyme activity, or metabolic activity (Douglas et al., 2020).

## 3 Results

### 3.1 Effects of microbial treatment on fruit set date and soluble solids content

The microbial treatment significantly advanced blueberry fruit set compared with the untreated control (Figure 1). The fruit set date was 57.56 ± 1.33 DAT in NT and 46.67 ± 2.06 DAT in BT. Thus, fruit set in BT occurred approximately 10.89 days earlier than in NT. Welch’s two-sample t-test indicated a highly significant difference between treatments (*t* = 13.31, df = 13.70, *p* = 3.21 × 10^−9^). The soluble solids content of blueberry fruit was significantly higher in the BT treatment than in the NT treatment. The SSC values were 11.33 ± 1.00 °Brix for NT and 15.00 ± 0.71 °Brix for BT. Therefore, BT increased SSC by 3.67 °Brix relative to NT. This difference was highly significant according to Welch’s two-sample t-test (*t* = −8.98, df = 14.40, *p* = 2.78 × 10^−7^). No apparent differences were observed between NT and BT in other measured plant growth-related traits, including plant size, during the experimental period (data not shown).

**Figure 1:**
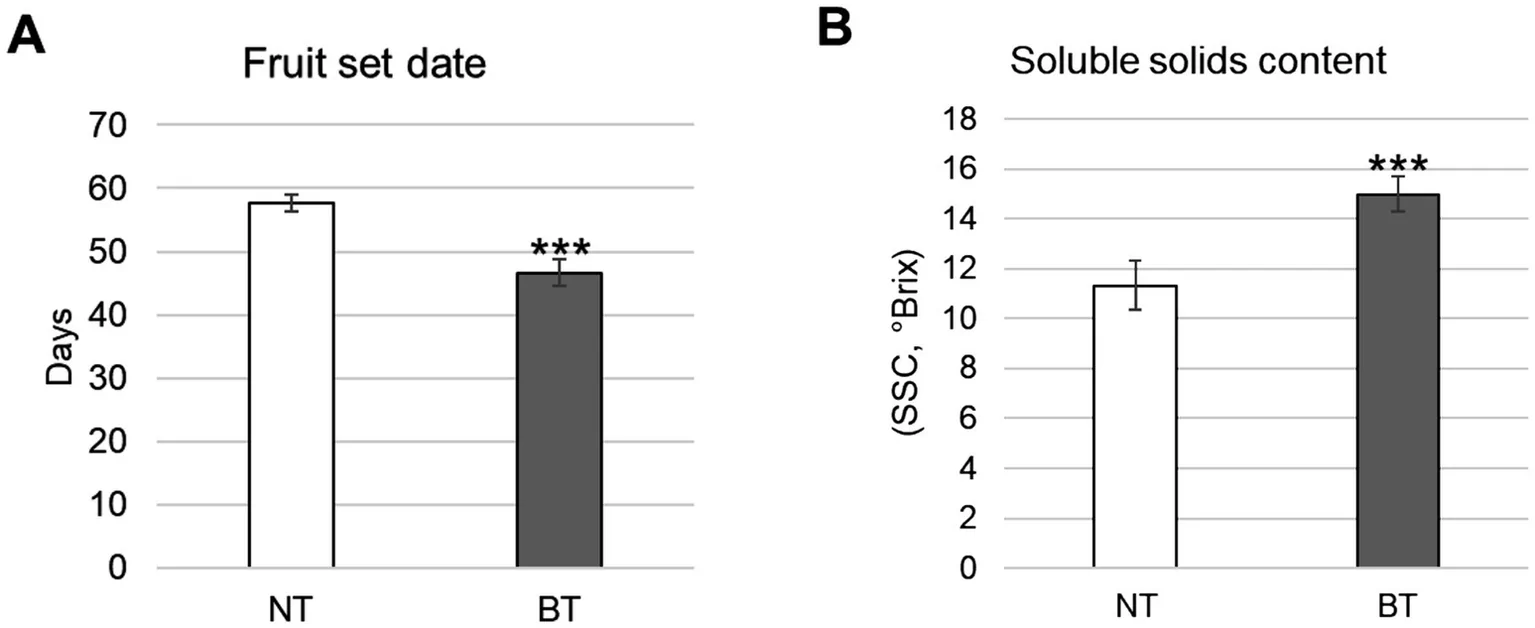
Effects of microbial treatment on blueberry fruit set date and soluble solids content. NT, untreated control; BT, microbial treatment. **(A)** Fruit set date, expressed as days after treatment initiation (DAT). **(B)** Soluble solids content (SSC), expressed as °Brix. Bars represent the mean ± standard deviation of nine individual biological replicates per treatment (*n* = 9). Differences between NT and BT were evaluated using Welch’s two-sample *t*-test. Asterisks indicate statistical significance: *p < 0.05, **p < 0.01, ***p < 0.001.

### 3.2 Soil chemical properties under different treatments

Soil chemical properties differed between NT and BT at the end of the cultivation period (Table 1). Electrical conductivity (EC) was significantly lower in BT (1.57 ± 0.09 dS m^−1^) than in NT (3.54 ± 0.08 dS m^−1^) (*p* < 0.001). A similar pattern was observed for cation exchange capacity (CEC), which was lower in BT (24.21 ± 0.83 cmolc kg^−1^) than in NT (45.02 ± 1.62 cmolc kg^−1^) (*p* < 0.001).

**Table 1:** Soil chemical properties of untreated and microbial culture-treated blueberry rhizosphere soils at harvest.

Property	NT	BT	ANOVA P
pH [1:5]	5.18 ± 0.05	5.20 ± 0.10	0.8121
EC [1:1] (dS m^−1^)	3.54 ± 0.08	1.57 ± 0.09	0.0000***
CEC (cmolc kg^−1^)	45.02 ± 1.62	24.21 ± 0.83	0.0000 ***
T-N (%)	0.68 ± 0.00	0.28 ± 0.01	0.0000***
A-P (mg kg^−1^)	364.57 ± 14.50	722.70 ± 118.28	0.0065**
K (cmolc kg^−1^)	0.37 ± 0.07	0.13 ± 0.02	0.0058**
Ca (cmolc kg^−1^)	21.30 ± 0.66	9.85 ± 0.90	0.0001***
Mg (cmolc kg^−1^)	1.69 ± 0.08	1.07 ± 0.11	0.0016**
Na (cmolc kg^−1^)	0.05 ± 0.00	0.06 ± 0.01	0.1583
Salinity (%)	0 ± 0	0 ± 0	0.3739
OM (%)	28.04 ± 3.06	11.38 ± 1.22	0.0009***
NH₄^+^–N (mg kg^−1^)	144.77 ± 29.16	84.06 ± 14.01	0.0314*
NO₃^−^–N (mg kg^−1^)	275.53 ± 56.62	275.53 ± 21.40	1.0000

NT, the control treatment; BT, the microbial treatment. Values are presented as the mean ± standard deviation of three individual biological replicates per treatment (*n* = 3). Statistical differences between NT and BT were evaluated using one-way analysis of variance (ANOVA). Asterisks indicate significant differences between treatments: *p < 0.05, **p < 0.01, and ***p < 0.001.

Nitrogen-related soil properties also differed between treatments. Total nitrogen (T-N) was significantly lower in BT (0.28 ± 0.01%) than in NT (0.68 ± 0.00%) (*p* < 0.001). Ammonium nitrogen (NH₄^+^–N) showed a similar pattern, with concentrations of 84.06 ± 14.01 mg kg^−1^ in BT and 144.77 ± 29.16 mg kg^−1^ in NT (*p* = 0.0314). In contrast, nitrate nitrogen (NO₃^−^–N) did not differ significantly between treatments, indicating that the observed differences were not consistent across inorganic nitrogen forms.

Exchangeable cation concentrations also differed between treatments. Exchangeable potassium (K) was lower in BT (0.13 ± 0.02 cmolc kg^−1^) than in NT (0.37 ± 0.07 cmolc kg^−1^) (*p* = 0.0058). Exchangeable calcium (Ca) decreased from 21.30 ± 0.66 cmolc kg^−1^ in NT to 9.85 ± 0.90 cmolc kg^−1^ in BT (*p* < 0.001), while exchangeable magnesium (Mg) was 1.69 ± 0.08 cmolc kg^−1^ in NT and 1.07 ± 0.11 cmolc kg^−1^ in BT (*p* = 0.0016).

Organic matter (OM) content was significantly lower in BT (11.38 ± 1.22%) than in NT (28.04 ± 3.06%) (*p* = 0.0009). In contrast to the lower values observed for several other soil chemical properties, available phosphorus (A-P) was significantly higher in BT (722.70 ± 118.28 mg kg^−1^) than in NT (364.57 ± 14.50 mg kg^−1^) (*p* = 0.0065).

Several soil properties did not differ significantly between treatments. Soil pH was similar between NT (5.18 ± 0.05) and BT (5.20 ± 0.10) (*p* = 0.8121). Exchangeable Na, salinity, and NO₃^−^–N also showed no significant differences between treatments. Overall, compared with NT, BT was characterized by lower EC, CEC, T-N, NH₄^+^–N, exchangeable K, Ca, and Mg, and OM, together with higher available P at the end of the cultivation period.

### 3.3 Correlation structure among soil chemical properties

To explore patterns of covariation among soil chemical properties, Pearson’s correlation analysis was performed across all six biological replicate samples (three NT and three BT samples) (Table 2). Because treatment was not included as a covariate and the sample size was limited, the correlations were interpreted as exploratory treatment-associated patterns rather than as intrinsic relationships among soil properties.

**Table 2:** Pearson correlation coefficients among soil chemical properties.

Parameters	pH	EC	CEC	T-N	A-P	K	Ca	Mg	Na	Salinity	OM	NH₄^+^–N	NO₃^−^–N
pH	1	−0.110	−0.059	−0.144	0.399	−0.063	−0.121	−0.137	0.346	0.687	−0.017	−0.087	0.007
EC	−0.110	1	0.997***	0.997***	−0.916*	0.953**	0.998***	0.981***	−0.613	−0.405	0.983***	0.863*	−0.028
CEC	−0.059	0.997***	1	0.993***	−0.911*	0.960**	0.990***	0.969**	−0.628	−0.406	0.991***	0.864*	−0.040
T-N	−0.144	0.997***	0.993***	1	−0.939**	0.933**	0.993***	0.966**	−0.661	−0.46	0.971**	0.848*	0.007
A-P	0.399	−0.916*	−0.911*	−0.939**	1	−0.853*	−0.904*	−0.859*	0.836*	0.735	−0.881*	−0.803	0.038
K	−0.063	0.953**	0.96**	0.933**	−0.853*	1	0.944**	0.956**	−0.561	−0.359	0.986***	0.926**	−0.274
Ca	−0.121	0.998***	0.990***	0.993***	−0.904*	0.944**	1	0.988***	−0.583	−0.374	0.974***	0.861*	−0.023
Mg	−0.137	0.981***	0.969**	0.966**	−0.859*	0.956**	0.988***	1	−0.483	−0.302	0.965**	0.861*	−0.074
Na	0.346	−0.613	−0.628	−0.661	0.836*	−0.561	−0.583	−0.483	1	0.878*	−0.601	−0.643	0.175
Salinity	0.687	−0.405	−0.406	−0.460	0.735	−0.359	−0.374	−0.302	0.878*	1	−0.368	−0.381	0.060
OM	−0.017	0.983***	0.991***	0.971**	−0.881*	0.986***	0.974***	0.965**	−0.601	−0.368	1	0.903*	−0.153
NH₄^+^–N	−0.087	0.863*	0.864*	0.848*	−0.803	0.926**	0.861*	0.861*	−0.643	−0.381	0.903*	1	−0.517
NO₃^−^–N	0.007	−0.028	−0.040	0.007	0.038	−0.274	−0.023	−0.074	0.175	0.060	−0.153	−0.517	1

NT, the control treatment; BT, the microbial treatment. Pearson correlation coefficients were calculated among soil chemical properties using all six biological samples (NT, *n* = 3; BT, *n* = 3). Correlations were calculated across pooled NT and BT samples without adjustment for treatment effects and were therefore interpreted as exploratory treatment-associated covariation. Asterisks indicate nominal significance levels: *p < 0.05, **p < 0.01, and ***p < 0.001.

Electrical conductivity (EC) showed strong positive correlations with cation exchange capacity (CEC) (*r* = 0.997, *p* < 0.001), total nitrogen (T-N) (*r* = 0.997, *p* < 0.001), exchangeable calcium (Ca) (*r* = 0.998, *p* < 0.001), exchangeable magnesium (Mg) (*r* = 0.981, *p* < 0.001), and organic matter (OM) (*r* = 0.983, *p* < 0.001). Similarly, CEC was positively correlated with T-N (*r* = 0.993, *p* < 0.001), Ca (*r* = 0.990, *p* < 0.001), Mg (*r* = 0.969, *p* < 0.01), and OM (*r* = 0.991, *p* < 0.001). T-N also showed positive correlations with Ca (*r* = 0.993, *p* < 0.001), Mg (*r* = 0.966, *p* < 0.01), and OM (*r* = 0.971, *p* < 0.01), indicating coordinated covariation among these soil chemical properties across the six samples.

Exchangeable potassium (K) showed similar positive relationships with CEC (*r* = 0.960, *p* < 0.01), T-N (*r* = 0.933, *p* < 0.01), Ca (*r* = 0.944, *p* < 0.01), Mg (*r* = 0.956, *p* < 0.01), and OM (*r* = 0.986, *p* < 0.001). Ammonium nitrogen (NH₄^+^–N) was also positively correlated with EC (*r* = 0.863, *p* < 0.05), CEC (*r* = 0.864, *p* < 0.05), T-N (*r* = 0.848, *p* < 0.05), K (*r* = 0.926, *p* < 0.01), and OM (*r* = 0.903, *p* < 0.05).

In contrast, available phosphorus (A-P) showed an inverse pattern, with negative correlations with EC (*r* = −0.916, *p* < 0.05), CEC (*r* = −0.911, *p* < 0.05), T-N (*r* = −0.939, *p* < 0.01), Ca (*r* = −0.904, *p* < 0.05), Mg (*r* = −0.859, *p* < 0.05), and OM (*r* = −0.881, *p* < 0.05). These patterns indicate that A-P varied in the opposite direction to several soil properties that were higher in NT than in BT.

Nitrate nitrogen (NO₃^−^–N) showed weak correlations with most soil chemical properties, including EC (*r* = −0.028), CEC (*r* = −0.040), and T-N (*r* = 0.007). Soil pH also showed relatively limited correlations with most variables, although correlations of moderate magnitude were observed with salinity (*r* = 0.687) and A-P (*r* = 0.399). Exchangeable sodium (Na) was positively correlated with A-P (*r* = 0.836, *p* < 0.05) and salinity (*r* = 0.878, *p* < 0.05), while weaker associations were observed with the remaining variables.

Overall, the analysis revealed coordinated covariation among EC, CEC, T-N, exchangeable cations, OM, and NH₄^+^–N, together with an opposing pattern for available P. However, because the analysis was based on only six biological samples and treatment effects were not statistically separated from the correlations, these relationships should be interpreted cautiously as exploratory treatment-associated covariation. In particular, some of the observed correlations may partly reflect the overall chemical separation between NT and BT rather than direct or intrinsic relationships among individual soil properties.

### 3.4 Bacterial community structure based on 16S rRNA sequencing

Bacterial community structure in NT and BT rhizosphere soils was examined using the final quality-controlled, pre-clustered bacterial sequence-feature dataset obtained from full-length 16S rRNA gene sequencing. Rarefaction analysis showed increasing numbers of observed bacterial sequence features with increasing sequencing depth across all six biological samples (Figure 2A). Sequencing depth varied among samples, with NT_3 exhibiting the greatest read depth, whereas the remaining samples were distributed within a broadly comparable range. A common rarefaction depth was therefore applied to retain all six samples for standardized community comparisons.

**Figure 2:**
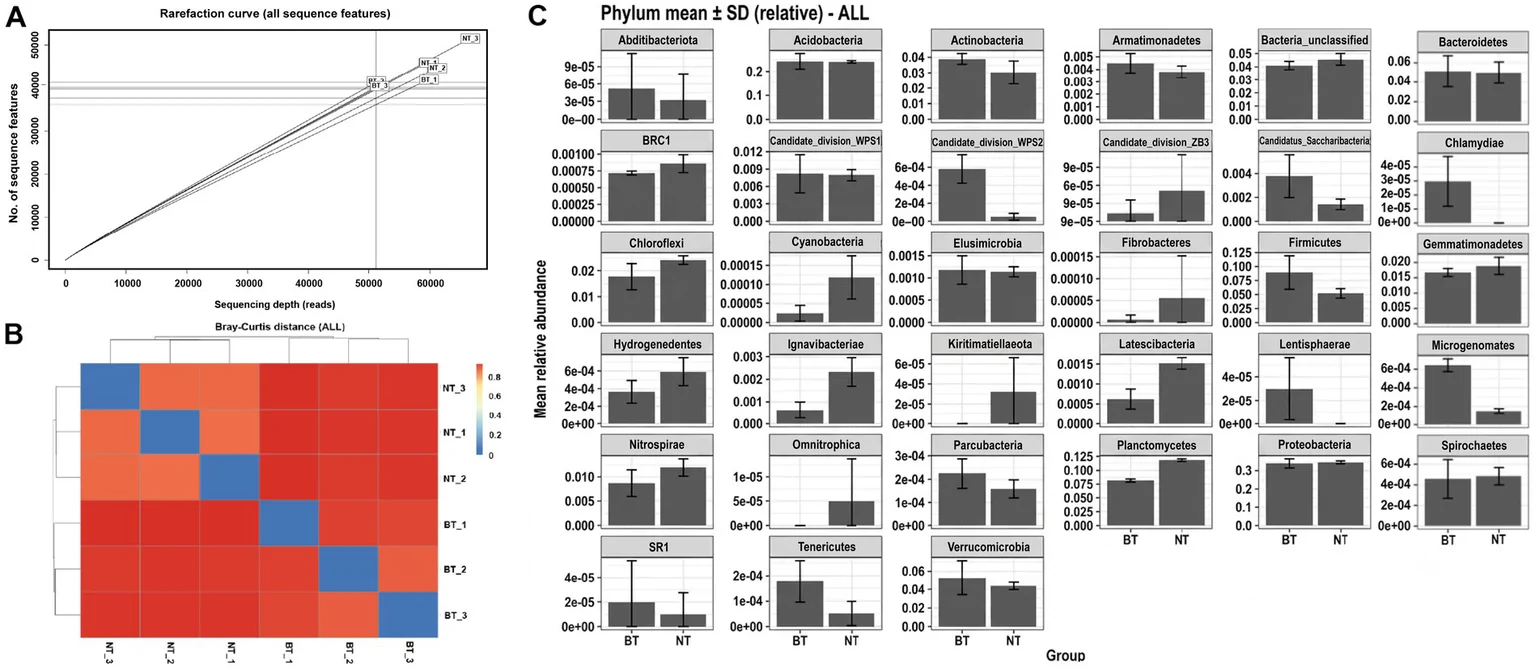
Bacterial community structure in untreated and microbial-treated blueberry soils. NT, the control treatment; BT, the microbial treatment. **(A)** Rarefaction curves showing the accumulation of observed quality-controlled, pre-clustered bacterial sequence features with increasing sequencing depth for each individual rhizosphere soil sample. **(B)** Bray–Curtis dissimilarity heatmap with hierarchical clustering of bacterial communities. Lower and higher dissimilarity values are represented by blue and red colors, respectively. Differences in overall bacterial community composition between NT and BT were evaluated by PERMANOVA (pseudo-*F* = 1.464, *R*^2^ = 0.268, *p* = 0.100). **(C)** Mean relative abundances of bacterial phyla in NT and BT. Bars represent the mean relative abundance and error bars indicate the standard deviation of three individual biological replicates per treatment (*n* = 3).

The rarefaction curves did not reach a complete asymptote within the observed sequencing range, indicating that additional sequencing could potentially recover further bacterial sequence features. Nevertheless, all six samples provided sufficient reads for comparison at the common standardized depth, and no sample was excluded on the basis of sequencing depth. Accordingly, subsequent standardized community comparisons were conducted using the same rarefaction depth across NT and BT samples.

Bray–Curtis dissimilarity showed an apparent treatment-associated clustering pattern between NT and BT samples (Figure 2B). In the hierarchical clustering based on Bray–Curtis dissimilarity, the three NT samples clustered together and were separated from the three BT samples. However, formal comparison by PERMANOVA did not detect a statistically significant difference in overall bacterial community composition between treatments (pseudo-*F* = 1.464, R^2^ = 0.268, *p* = 0.100). Thus, although the Bray–Curtis pattern suggested treatment-associated differentiation, the separation was not statistically supported and was interpreted as exploratory rather than as evidence of a confirmed treatment effect on overall bacterial community structure.

At the phylum level, Proteobacteria and Acidobacteria were the dominant bacterial groups in both NT and BT rhizosphere soils (Figure 2C), indicating broadly similar dominant phylum-level composition between treatments. Differences in mean relative abundance were nevertheless observed for several bacterial phyla. Firmicutes, Actinobacteria, Verrucomicrobia, Microgenomates, Parcubacteria, Tenericutes, Chlamydiae, and Candidatus Saccharibacteria showed higher mean relative abundance in BT than in NT. In contrast, Planctomycetes, Chloroflexi, Nitrospirae, Latescibacteria, Ignavibacteriae, and several low-abundance phyla showed higher mean relative abundance in NT. Other dominant or moderately abundant groups, including Acidobacteria and Bacteroidetes, showed relatively small differences in mean relative abundance between treatments.

Overall, the dominant phylum-level composition remained broadly similar between NT and BT, while several bacterial groups showed treatment-associated differences in mean relative abundance. Because these phylum-level patterns were based on descriptive relative-abundance comparisons among three biological replicates per treatment, they were interpreted as compositional trends rather than as evidence of statistically confirmed differential abundance.

### 3.5 Diversity, taxonomic composition, and environmental associations of treatment-responsive bacterial sequence features

The union of bacterial sequence features identified as treatment responsive between NT and BT was further evaluated to characterize their diversity, taxonomic composition, and associations with rhizosphere soil chemical properties (Figure 3; Table 3). Within this treatment-responsive sequence-feature subset, observed richness, Shannon diversity, and Simpson diversity were consistently higher in BT than in NT (Figure 3A). All three BT biological replicates showed greater observed richness and higher Shannon and Simpson diversity indices than the NT replicates. These patterns indicate greater diversity within the treatment-responsive bacterial sequence-feature subset in BT. However, because these indices were calculated from bacterial sequence features selected on the basis of treatment responsiveness, they describe diversity patterns within the selected subset and should not be interpreted as measures of total bacterial community diversity.

**Figure 3:**
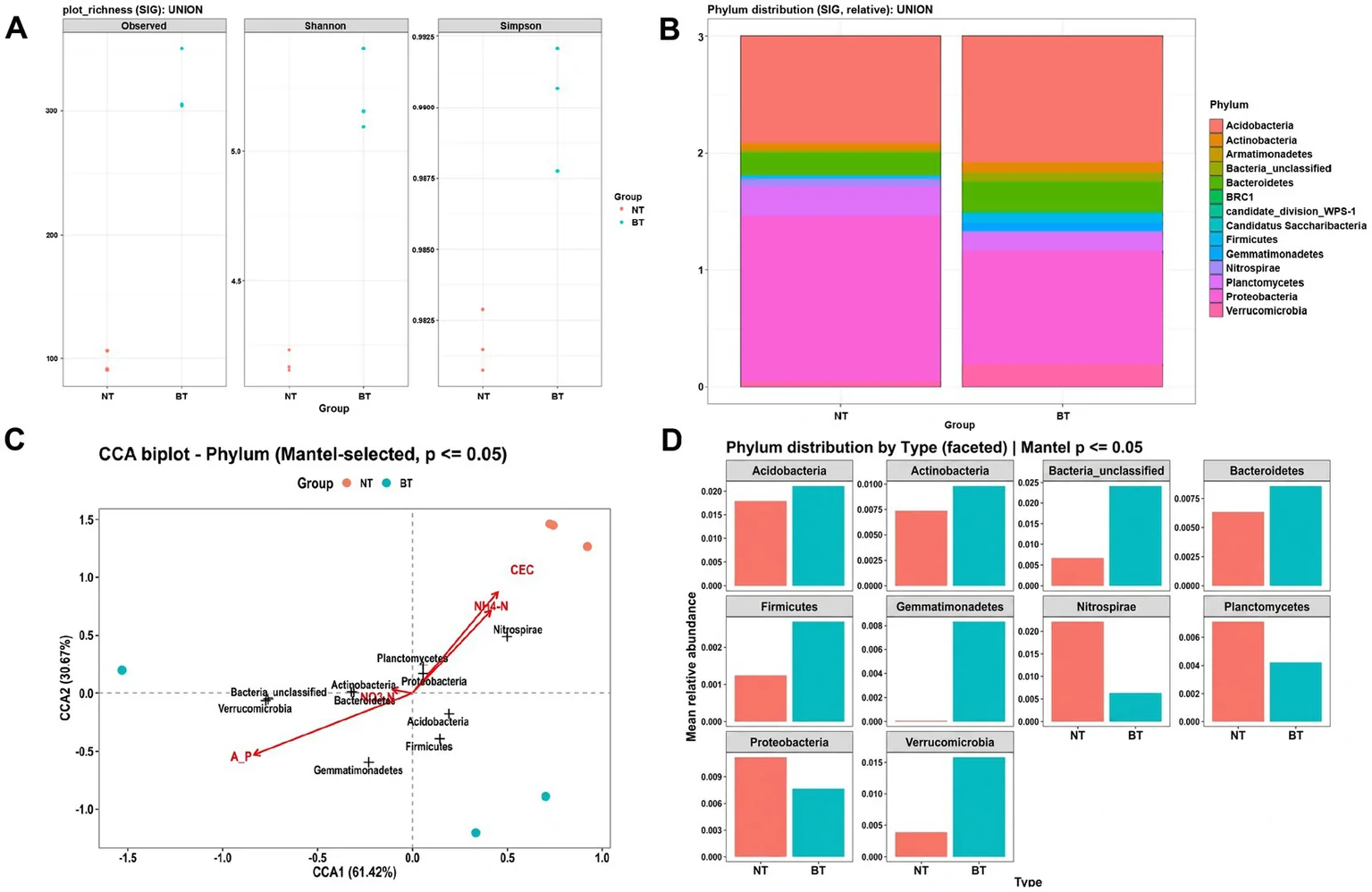
Diversity, phylum-level composition, and environmental associations of treatment-responsive bacterial sequence features in blueberry-cultivated soil. **(A)** Alpha diversity of the union of treatment-responsive bacterial sequence features identified between NT and BT. Observed richness, Shannon diversity, and Simpson diversity are shown for individual biological replicates. **(B)** Phylum-level relative abundance profiles of the treatment-responsive bacterial sequence-feature subset in NT and BT. **(C)** Canonical correspondence analysis (CCA) biplot showing the multivariate covariation among treatment-responsive bacterial phyla, soil chemical properties, and soil samples. Fitted soil chemical variables are indicated by red arrows. **(D)** Mean relative abundances of bacterial phyla retained by exploratory Mantel screening at nominal *p* ≤ 0.05. Bars represent mean relative abundance, and error bars indicate standard deviation. NT, the control treatment; BT, the microbial treatment; A-P, available phosphorus; NO₃^−^-N, nitrate nitrogen; NH₄^+^-N, ammonium nitrogen; CEC, cation exchange capacity; EC, electrical conductivity; T-N, total nitrogen; Ca, exchangeable calcium; Mg, exchangeable magnesium; Na, exchangeable sodium.

**Table 3:** Exploratory Mantel associations between treatment-responsive bacterial phyla and soil chemical properties in blueberry-cultivated soil.

Taxonomic_Group	Environmental_Factor	Mantel_r	p_value
Acidobacteria	EC	0.629	0.0097**
	Ca	0.611	0.0125*
	OM	0.604	0.0181*
	Mg	0.550	0.0208*
	CEC	0.518	0.0486*
Actinobacteria	T-N	0.750	0.0014**
	A-P	0.729	0.0042**
Bacteria_unclassified	A-P	0.757	0.0042**
	T-N	0.671	0.0011**
Bacteroidetes	A-P	0.768	0.0042**
	T-N	0.714	0.0056**
Firmicutes	Ca	0.925	0.0028**
	EC	0.846	0.0125*
	Mg	0.836	0.0139*
	NH₄^+^–N	0.638	0.0278*
	K	0.761	0.0361*
	OM	0.729	0.0431*
Gemmatimonadetes	Mg	0.606	0.0111*
	EC	0.539	0.0139*
	Ca	0.534	0.0139*
	T-N	0.382	0.0472*
Nitrospirae	NH₄^+^–N	0.914	0.0028**
	K	0.921	0.0056**
	OM	0.850	0.0111*
	EC	0.825	0.0139*
	CEC	0.821	0.0139*
	Mg	0.800	0.0194*
	Ca	0.729	0.0361*
Planctomycetes	T-N	0.486	0.0458*
	A-P	0.461	0.0486*
Proteobacteria	A-P	0.929	0.0028**
	NH₄^+^–N	0.706	0.0111*
	T-N	0.818	0.0153*
	Na	0.480	0.0333*
	Ca	0.729	0.0417*
	K	0.718	0.0486*
Verrucomicrobia	A-P	0.707	0.0069**
	T-N	0.593	0.0097**

Mantel r indicates the strength and direction of the association between the Euclidean distance matrix derived from the phylum-specific relative-abundance profile and the Euclidean distance matrix derived from each soil chemical property. Only associations with nominal Mantel *p* ≤ 0.05 are presented. Mantel tests were used as an exploratory screening procedure, and p-values were not adjusted for multiple comparisons at this stage; therefore, the reported associations should be interpreted as exploratory rather than as confirmatory statistical evidence. A-P, available phosphorus; T-N, total nitrogen; EC, electrical conductivity; CEC, cation exchange capacity; OM, organic matter; NH₄^+^–N, ammonium nitrogen; K, exchangeable potassium; Ca, exchangeable calcium; Mg, exchangeable magnesium; Na, exchangeable sodium. Asterisks indicate nominal p-value levels: *p < 0.05, **p < 0.01, and ***p < 0.001.

The phylum-level composition of the treatment-responsive sequence-feature subset showed descriptive differences between NT and BT (Figure 3B). Proteobacteria and Acidobacteria represented major components of the selected sequence-feature profiles in both treatments, while the relative contributions of several other bacterial phyla differed between groups. Actinobacteria, Bacteroidetes, Firmicutes, Gemmatimonadetes, Verrucomicrobia, Planctomycetes, Nitrospirae, and several less abundant bacterial groups contributed differently to the NT and BT profiles. These patterns indicate treatment-associated differences in the taxonomic distribution of the selected sequence features rather than a statistically confirmed shift in the entire bacterial community.

Canonical correspondence analysis (CCA) was subsequently used to explore covariation between the Mantel-selected bacterial phyla and rhizosphere soil chemical properties (Figure 3C). CCA1 accounted for 61.42% of the constrained variation and CCA2 accounted for 30.67%, with the first two axes together representing 92.09% of the constrained variation displayed in the ordination. The NT samples were positioned primarily in the positive region of CCA1 and CCA2, whereas the BT samples were distributed away from the NT cluster, primarily toward negative CCA2 or negative CCA1 scores. Cation exchange capacity (CEC) and ammonium nitrogen (NH₄^+^–N) were oriented toward the positive directions of CCA1 and CCA2, whereas available phosphorus (A-P) was strongly oriented toward the negative direction of CCA1. The NO₃^−^–N vector was comparatively short and positioned near the origin. Nitrospirae was located broadly in the direction of the CEC and NH₄^+^–N vectors, whereas Verrucomicrobia and unclassified bacterial groups were positioned toward the negative region of CCA1, broadly corresponding to the direction of A-P. Gemmatimonadetes was positioned toward negative CCA1 and CCA2, whereas Acidobacteria and Firmicutes were located primarily toward positive CCA1 and negative CCA2. Given the limited number of biological replicates, these CCA patterns were interpreted as exploratory covariation rather than as evidence of direct environmental effects.

Phylum-specific comparisons of the Mantel-selected bacterial groups further showed treatment-associated abundance patterns (Figure 3D). Acidobacteria, Actinobacteria, Bacteria_unclassified, Bacteroidetes, Firmicutes, Gemmatimonadetes, and Verrucomicrobia showed higher mean relative abundances in BT than in NT. In contrast, Nitrospirae, Planctomycetes, and Proteobacteria showed higher mean relative abundances in NT than in BT. The differences in mean relative abundance were particularly pronounced for Bacteria_unclassified, Gemmatimonadetes, Verrucomicrobia, and Nitrospirae. These comparisons were interpreted as descriptive abundance patterns because the plotted differences were not, by themselves, evidence of statistically significant differential abundance.

Exploratory Mantel analyses identified 38 nominal phylum–soil property associations across 10 bacterial phyla (*p* ≤ 0.05; Table 3). Acidobacteria showed positive associations with EC (*r* = 0.629, *p* = 0.0097), Ca (*r* = 0.611, *p* = 0.0125), OM (*r* = 0.604, *p* = 0.0181), Mg (*r* = 0.550, *p* = 0.0208), and CEC (*r* = 0.518, *p* = 0.0486). Actinobacteria was associated with T-N (*r* = 0.750, *p* = 0.0014) and A-P (*r* = 0.729, *p* = 0.0042), while Bacteria_unclassified showed associations with A-P (*r* = 0.757, *p* = 0.0042) and T-N (*r* = 0.671, *p* = 0.0011). Bacteroidetes was similarly associated with A-P (*r* = 0.768, *p* = 0.0042) and T-N (*r* = 0.714, *p* = 0.0056).

Firmicutes showed associations with Ca (*r* = 0.925, *p* = 0.0028), EC (*r* = 0.846, *p* = 0.0125), Mg (*r* = 0.836, *p* = 0.0139), NH₄^+^–N (*r* = 0.638, *p* = 0.0278), K (*r* = 0.761, *p* = 0.0361), and OM (*r* = 0.729, *p* = 0.0431). Gemmatimonadetes showed nominal associations with Mg (*r* = 0.606, *p* = 0.0111), EC (*r* = 0.539, *p* = 0.0139), Ca (*r* = 0.534, *p* = 0.0139), and T-N (*r* = 0.382, *p* = 0.0472). Nitrospirae exhibited associations with NH₄^+^–N (*r* = 0.914, *p* = 0.0028), K (*r* = 0.921, *p* = 0.0056), OM (*r* = 0.850, *p* = 0.0111), EC (*r* = 0.825, *p* = 0.0139), CEC (*r* = 0.821, *p* = 0.0139), Mg (*r* = 0.800, *p* = 0.0194), and Ca (*r* = 0.729, *p* = 0.0361). Planctomycetes showed comparatively weaker nominal associations with T-N (*r* = 0.486, *p* = 0.0458) and A-P (*r* = 0.461, *p* = 0.0486).

Proteobacteria showed associations with A-P (*r* = 0.929, *p* = 0.0028), NH₄^+^–N (r = 0.706, *p* = 0.0111), T-N (*r* = 0.818, *p* = 0.0153), Na (*r* = 0.480, *p* = 0.0333), Ca (*r* = 0.729, *p* = 0.0417), and K (*r* = 0.718, *p* = 0.0486). Verrucomicrobia showed associations with A-P (*r* = 0.707, *p* = 0.0069) and T-N (*r* = 0.593, *p* = 0.0097). Among the nominal associations, the largest Mantel correlation coefficient was observed between Proteobacteria and A-P (*r* = 0.929), followed by Firmicutes and Ca (*r* = 0.925), Nitrospirae and K (*r* = 0.921), and Nitrospirae and NH₄^+^–N (*r* = 0.914). Because multiple Mantel tests were conducted without multiple-testing correction, these relationships were interpreted as exploratory associations rather than confirmatory evidence of specific soil chemical effects on bacterial taxa.

### 3.6 Genus-level treatment-associated abundance patterns of bacterial communities

Genus-level exploratory analysis identified 68 bacterial genera or unclassified taxonomic groups belonging to eight phyla that showed nominal treatment-associated abundance patterns between NT and BT (Table 4). These taxa were retained based on Spearman correlations between treatment group and genus-level relative abundance at a nominal threshold of *p* < 0.05. Positive Spearman correlations indicated taxa with higher mean relative abundance in BT, whereas negative correlations indicated taxa with higher mean relative abundance in NT. Because no multiple-testing correction was applied, these genus-level patterns were interpreted as exploratory treatment-associated associations rather than as statistically confirmed differential abundance.

**Table 4:** Exploratory genus-level treatment-associated abundance patterns between untreated (NT) and microbial-treated (BT) blueberry rhizosphere soils.

Phylum	Genus	MeanRelAb_NT	MeanRelAb_BT	Log2FC_BT_vs_NT	SpearmanR_Group_vs_Abundance	SpearmanP_Group_vs_Abundance
Acidobacteria	Blastocatellales_unclassified	0.040	0	−35.221	−0.933	0.007
	Gp22_unclassified	0.045	0	−35.390	−0.933	0.007
	Acidobacteria_Gp1_unclassified	0	0.019	34.125	0.933	0.007
	Acidobacteria_Gp3_unclassified	0	0.025	34.550	0.933	0.007
	Edaphobacter_unclassified	0	0.025	34.546	0.933	0.007
	Gp17_unclassified	0	0.005	32.178	0.933	0.007
	Occallatibacter_unclassified	0	0.055	35.677	0.933	0.007
	Candidatus_Solibacter_unclassified	0.003	0.060	4.405	0.891	0.017
	Gp4_unclassified	0.201	0.001	−8.063	−0.891	0.017
	Gp1_unclassified	0.110	0.270	1.294	0.878	0.021
	Gp2_unclassified	0.083	0.262	1.653	0.878	0.021
	Gp6_unclassified	0.359	0.079	−2.184	−0.878	0.021
Actinobacteria	Gaiella	0.004	0.679	7.383	0.891	0.017
	Solirubrobacterales_unclassified	0.996	0.264	−1.915	−0.891	0.017
Bacteroidetes	Cytophagales_unclassified	0	0.167	37.278	0.933	0.007
	Fulvivirgaceae_unclassified	0	0.013	33.621	0.933	0.007
	Puia	0	0.016	33.858	0.933	0.007
	Flavitalea	0.029	0.719	4.638	0.878	0.021
	Ferruginibacter	0.101	0.030	−1.780	−0.878	0.021
	Terrimonas	0.867	0.004	−7.929	−0.878	0.021
Firmicutes	Paenibacillaceae_1_unclassified	0.992	0	−39.851	−0.949	0.004
	Bacillaceae_2_unclassified	0	0.185	37.432	0.933	0.007
	Bacillales_unclassified	0	0.231	37.750	0.933	0.007
	Bacillus	0	0.214	37.642	0.933	0.007
	Clostridium_sensu_stricto	0	0.100	36.542	0.933	0.007
Gemmatimonadetes	Roseisolibacter	0	0.414	38.594	0.933	0.007
Planctomycetes	Lacipirellula	0.124	0	−36.853	−0.933	0.007
	Planctomicrobium	0.108	0	−36.649	−0.933	0.007
	Isosphaeraceae_unclassified	0	0.028	34.717	0.933	0.007
	Planctomycetaceae_unclassified	0	0.102	36.570	0.933	0.007
	Planctomycetales_unclassified	0	0.035	35.020	0.933	0.007
	Singulisphaera	0	0.012	33.5323	0.933	0.007
	Thermoguttaceae_unclassified	0.199	0.089	−1.161	−0.878	0.021
Proteobacteria	Rhodomicrobium	0.053	0	−35.637	−0.933	0.007
	Rhodoplanes	0.013	0	−33.647	−0.933	0.007
	Rhodospirillaceae_unclassified	0.066	0	−35.934	−0.933	0.007
	Acetobacteraceae_unclassified	0	0.022	34.370	0.933	0.007
	Acidibacter_unclassified	0	0.036	35.081	0.933	0.007
	Bauldia	0	0.006	32.521	0.933	0.007
	Bradyrhizobiaceae_unclassified	0	0.133	36.957	0.933	0.007
	Hyphomicrobiaceae_unclassified	0	0.021	34.338	0.933	0.007
	Labilithrix	0	0.002	31.097	0.933	0.007
	Methyloligella	0	0.006	32.375	0.933	0.007
	Myxococcales_unclassified	0	0.021	34.308	0.933	0.007
	Reyranella	0	0.009	33.058	0.933	0.007
	Steroidobacter	0	0.021	34.325	0.933	0.007
	Xanthomonadaceae_unclassified	0	0.005	32.085	0.933	0.007
	Hyphomicrobium	3.27 × 10^−4^	0.021	5.994	0.891	0.017
	Methyloceanibacter	3.27 × 10^−4^	0.009	4.823	0.891	0.017
	Paraburkholderia	6.25 × 10^−4^	0.028	5.489	0.891	0.017
	Roseiarcus	0.001	0.018	4.289	0.891	0.017
	Geobacter	0.021	2.47 × 10^−4^	−6.384	−0.891	0.017
	Bradyrhizobium	0.002	0.128	6.348	0.878	0.021
	Pseudolabrys	0.109	0.221	1.025	0.878	0.021
	Rhizomicrobium	0.001	0.022	4.778	0.878	0.021
	Steroidobacteraceae_unclassified	0.001	0.007	3.124	0.878	0.021
	Alphaproteobacteria_unclassified	0.032	0.006	−2.507	−0.878	0.021
	Betaproteobacteria_unclassified	0.289	0.071	−2.031	−0.878	0.021
	Deltaproteobacteria_unclassified	0.047	0.024	−0.972	−0.878	0.021
	Dongia	0.101	0.011	−3.139	−0.878	0.021
	Gammaproteobacteria_unclassified	0.126	0.053	−1.248	−0.878	0.021
	Nitrosomonadales_unclassified	0.058	4.70 × 10^−4^	−6.952	−0.878	0.021
	Ramlibacter	0.024	0.003	−3.016	−0.878	0.021
	Rhizobiales_unclassified	0.031	4.70 × 10^−4^	−6.049	−0.878	0.021
Verrucomicrobia	Oleiharenicola	0	0.016	33.880	0.933	0.007
	Opitutus	0	0.044	35.371	0.933	0.007
	Subdivision3_unclassified	0.512	0.846	0.725	0.878	0.021
	Spartobacteria_unclassified	0.488	0.093	−2.383	−0.878	0.021

Within Acidobacteria, several taxa showed contrasting abundance patterns between treatments. Acidobacteria_Gp1_unclassified, Acidobacteria_Gp3_unclassified, Edaphobacter_unclassified, Gp17_unclassified, and Occallatibacter_unclassified were detected in BT but not in NT. Candidatus_Solibacter_unclassified, Gp1_unclassified, and Gp2_unclassified also showed higher mean relative abundances in BT, whereas Blastocatellales_unclassified, Gp22_unclassified, Gp4_unclassified, and Gp6_unclassified were more abundant in NT. Within Actinobacteria, Gaiella increased from 0.004 in NT to 0.679 in BT, whereas Solirubrobacterales_unclassified decreased from 0.996 in NT to 0.264 in BT.

Several Bacteroidetes taxa also showed pronounced treatment-associated patterns. Cytophagales_unclassified, Fulvivirgaceae_unclassified, and Puia were detected in BT but not in NT, while Flavitalea showed a marked increase from 0.029 in NT to 0.719 in BT. In contrast, Ferruginibacter and Terrimonas were more abundant in NT, with Terrimonas decreasing from 0.867 in NT to 0.004 in BT. Within Firmicutes, Bacillaceae_2_unclassified, Bacillales_unclassified, Bacillus, and Clostridium_sensu_stricto were detected in BT but not in NT, whereas Paenibacillaceae_1_unclassified showed the opposite pattern, decreasing from 0.992 in NT to undetectable levels in BT. The Gemmatimonadetes genus Roseisolibacter was also detected only in BT, with a mean relative abundance of 0.414.

Planctomycetes showed mixed treatment-associated responses. Lacipirellula and Planctomicrobium were detected in NT at mean relative abundances of 0.124 and 0.108, respectively, but were not detected in BT. In contrast, Isosphaeraceae_unclassified, Planctomycetaceae_unclassified, Planctomycetales_unclassified, and Singulisphaera were detected in BT but not in NT. Thermoguttaceae_unclassified was more abundant in NT (0.199) than in BT (0.089).

Proteobacteria contained the largest number of treatment-associated genus-level taxa. Several taxa, including Acetobacteraceae_unclassified, Acidibacter_unclassified, Bauldia, Bradyrhizobiaceae_unclassified, Hyphomicrobiaceae_unclassified, Labilithrix, Methyloligella, Myxococcales_unclassified, Reyranella, Steroidobacter, and Xanthomonadaceae_unclassified, were detected in BT but not in NT. Other taxa also showed higher abundance in BT, including Hyphomicrobium (3.27 × 10^−4^ in NT vs. 0.021 in BT), Methylocenibacter (3.27 × 10^−4^ vs. 0.009), Paraburkholderia (6.25 × 10^−4^ vs. 0.028), Roseiarcus (0.001 vs. 0.018), Bradyrhizobium (0.002 vs. 0.128), Pseudolabrys (0.109 vs. 0.221), and Rhizomicrobium (0.001 vs. 0.022).

In contrast, Rhodomicrobium, Rhodoplanes, and Rhodospirillaceae_unclassified were detected in NT but not in BT. Several additional Proteobacteria-related taxa were also more abundant in NT, including Geobacter, Alphaproteobacteria_unclassified, Betaproteobacteria_unclassified, Deltaproteobacteria_unclassified, Dongia, Gammaproteobacteria_unclassified, Nitrosomonadales_unclassified, Ramlibacter, and Rhizobiales_unclassified. Notably, Geobacter decreased from 0.021 in NT to 2.47 × 10^−4^ in BT, while Nitrosomonadales_unclassified decreased from 0.058 to 4.70 × 10^−4^.

Within Verrucomicrobia, Oleiharenicola and Opitutus were detected only in BT, at mean relative abundances of 0.016 and 0.044, respectively. Subdivision3_unclassified was also more abundant in BT (0.846) than in NT (0.512), whereas Spartobacteria_unclassified showed the opposite pattern, decreasing from 0.488 in NT to 0.093 in BT.

Across the 68 taxa listed in Table 4, Spearman correlation coefficients ranged from −0.949 to 0.933, with nominal *p*-values ranging from approximately 0.004 to 0.021. Overall, the genus-level analysis revealed contrasting treatment-associated abundance patterns across multiple bacterial lineages. BT was characterized by higher representation of several taxa within Acidobacteria, Bacteroidetes, Firmicutes, Gemmatimonadetes, Proteobacteria, and Verrucomicrobia, whereas other taxa within the same phyla showed higher abundance in NT. Given the limited number of biological replicates and the absence of multiple-testing correction, these patterns were interpreted as exploratory treatment-associated differences rather than as statistically confirmed differential abundance.

MeanRelAb_NT and MeanRelAb_BT indicate the mean relative abundance of each genus or unclassified taxonomic group in NT and BT, respectively. Log2FC_BT_vs_NT represents the log₂ fold change in mean relative abundance in BT relative to NT; positive and negative values indicate higher mean abundance in BT and NT, respectively. SpearmanR_Group_vs_Abundance and SpearmanP_Group_vs_Abundance represent the Spearman rank correlation coefficient and corresponding nominal *p*-value for the association between treatment group and genus-level relative abundance. Only taxa with nominal *p* < 0.05 are presented. No multiple-testing correction was applied; therefore, these associations should be interpreted as exploratory treatment-associated abundance patterns rather than as statistically confirmed differential abundance.

### 3.7 Predicted functional pathway profiles associated with treatment-responsive bacterial sequence features

Predicted functional profiles associated with the treatment-responsive bacterial sequence-feature subset differed descriptively between NT and BT (Figure 4). Among the 50 pathways displayed with higher predicted abundance in BT at the nominal *p* < 0.05 threshold, the largest difference was observed for pyruvate fermentation to isobutanol (PWY-7111), which showed the highest predicted abundance in BT among the displayed pathways. Several other highly represented pathways were related to amino acid biosynthesis and metabolism, including the superpathway of L-serine and glycine biosynthesis I, L-histidine biosynthesis, L-lysine biosynthesis I, L-methionine biosynthesis, the aspartate superpathway, and S-adenosyl-L-methionine biosynthesis and cycling.

**Figure 4:**
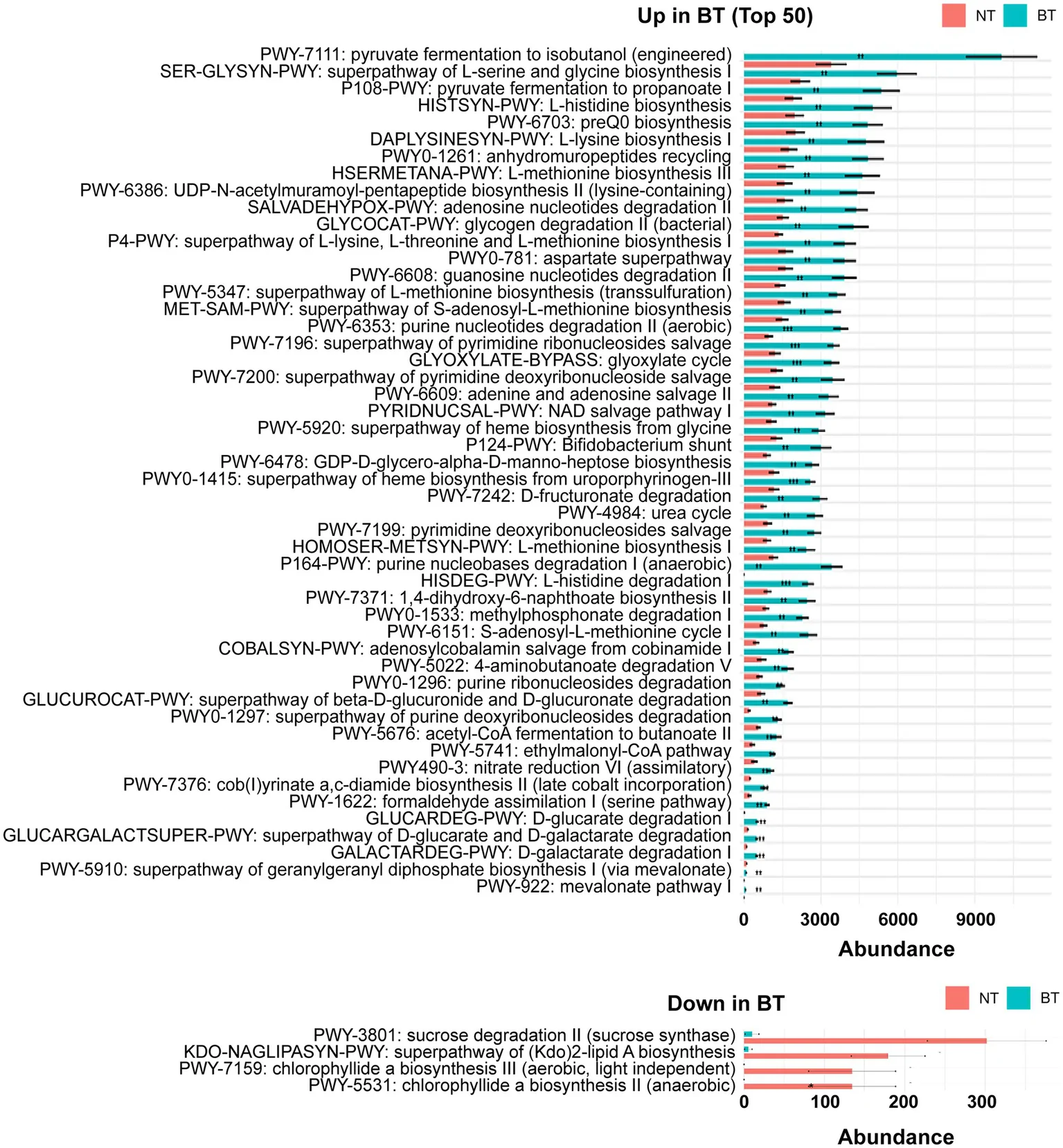
Predicted MetaCyc pathway profiles associated with treatment-responsive bacterial sequence features in untreated (NT) and microbial-treated (BT) blueberry rhizosphere soils. Pathways with higher predicted abundance in BT than in NT are shown in the upper panel, whereas pathways with lower predicted abundance in BT are shown in the lower panel. Each treatment comprised three individual-plant biological replicates (*n* = 3). Pathway-level comparisons were performed using two-sample t-tests at a nominal *p* < 0.05 threshold. No multiple-testing correction was applied; therefore, the displayed pathways represent exploratory treatment-associated differences in predicted functional potential rather than statistically confirmed functional changes. Functional profiles were inferred from 16S rRNA gene sequence data using PICRUSt2 and do not represent direct measurements of gene expression, enzyme activity, or microbial metabolic activity.

Pathways related to nucleotide metabolism were also frequently represented among those with higher predicted abundance in BT. These included adenosine and guanosine nucleotide degradation, purine nucleotide and nucleoside degradation, pyrimidine ribonucleoside and deoxyribonucleoside salvage, and adenine and adenosine salvage. Predicted pathways involved in carbohydrate utilization and central carbon metabolism were also represented, including glycogen degradation, the glyoxylate cycle, D-fructuronate degradation, β-D-glucuronide and D-glucuronate degradation, acetyl-CoA fermentation to butanoate, the ethylmalonyl-CoA pathway, and degradation pathways for D-glucarate and D-galactarate.

Several additional pathways with higher predicted abundance in BT were related to cofactor biosynthesis and salvage, cell-envelope-associated metabolism, and nitrogen-related functions. These included NAD salvage, heme biosynthesis, adenosylcobalamin salvage, cobyrinate biosynthesis, UDP-N-acetylmuramoyl-pentapeptide biosynthesis, GDP-D-glycero-α-D-manno-heptose biosynthesis, the urea cycle, and assimilatory nitrate reduction. Together, these patterns indicate that the selected bacterial sequence-feature subset in BT was associated with higher predicted representation of pathways spanning amino acid, nucleotide, carbon, cofactor, and nutrient-related metabolism.

In contrast, four pathways displayed lower predicted abundance in BT than in NT at the nominal *p* < 0.05 threshold: sucrose degradation II (sucrose synthase) (PWY-3801), the superpathway of (Kdo)₂-lipid A biosynthesis (KDO-NAGLIPASYN-PWY), chlorophyllide a biosynthesis III (aerobic, light-independent) (PWY-7159), and chlorophyllide a biosynthesis II (anaerobic) (PWY-5531). The two chlorophyllide biosynthesis pathways were detected predominantly in NT, while sucrose degradation II and the (Kdo)₂-lipid A biosynthesis superpathway also showed substantially higher predicted abundance in NT than in BT.

Overall, the exploratory PICRUSt2 analysis showed that the treatment-responsive bacterial sequence-feature subset was associated with broad differences in predicted functional potential between NT and BT, with substantially more displayed pathways showing higher predicted abundance in BT than lower predicted abundance. However, because pathway-level comparisons were based on nominal *p*-values without correction for multiple testing and functional profiles were inferred from 16S rRNA gene sequence information, these results should be interpreted as exploratory predictions of functional potential rather than as evidence of differences in gene abundance, gene expression, enzyme activity, or actual microbial metabolic activity.

## 4 Discussion

The present study showed that microbial treatment was associated with coordinated differences in blueberry fruit performance, rhizosphere soil chemistry, bacterial community composition, and predicted functional potential. Compared with NT, BT advanced the first visible fruit set by approximately 10.89 days and increased fruit soluble solids content by 3.67 °Brix, whereas no apparent differences were observed in vegetative growth-related traits, including plant size. This pattern suggests that the treatment was more strongly associated with reproductive development and fruit quality than with broad vegetative growth during the experimental period. Previous studies have reported that beneficial microbial consortia can improve blueberry production, fruit quality, soil nutrient conditions, and seedling performance (Schoebitz et al., 2016; Yu et al., 2020), although the magnitude and direction of these responses can depend on soil conditions, crop developmental stage, and inoculant composition (Backer et al., 2018; Philippot et al., 2013). The concurrent occurrence of earlier fruit set, higher soluble solids content, increased available phosphorus, and treatment-associated differences in the rhizosphere bacterial community suggests a possible linkage among fruit responses, rhizosphere nutrient status, and microbial community composition. Beneficial microorganisms can influence plant performance through nutrient solubilization, mineral transformation, phytohormone-related activity, and modification of indigenous rhizosphere communities (Compant et al., 2019; Richardson et al., 2009; Vessey, 2003). However, these relationships should be interpreted as associative because plant nutrient concentrations, phytohormones, photosynthetic activity, pollination success, fruit load, berry size, and ripening duration were not measured in the present study. Blueberry fruit set and soluble solids content are also influenced by cultivar, cultivation environment, and harvest stage; therefore, the fruit responses observed in the present unheated greenhouse experiment should be interpreted within the context of the ‘Duke’ cultivar and the defined experimental conditions (Cvetković et al., 2022; Kim et al., 2014). The higher SSC observed in BT is directionally consistent with the previous report that a beneficial bacterial consortium improved blueberry production, fruit quality, and soil properties under a different cultivation system (Yu et al., 2020). However, the inoculant composition and production conditions differed from those used here, and the present results do not demonstrate that identical mechanisms were responsible for the observed fruit responses.

At the end of the cultivation period, BT was characterized by lower EC, CEC, OM, total N, NH₄^+^–N, and exchangeable K, Ca, and Mg, whereas available P was substantially higher; soil pH, Na, salinity, and NO₃^−^–N did not differ significantly between treatments. This pattern indicates that the treatments were associated with contrasting distributions of measured soil nutrient pools and chemical properties rather than a uniform increase in soil fertility. Microorganisms can affect nutrient availability through organic matter decomposition, mineralization, immobilization, solubilization, plant–microbe competition, and changes in ion-exchange processes in the soil matrix (Le et al., 2025; Lee et al., 2025). The lower EC in BT indicates a lower concentration of soluble ions at the time of sampling, but the present measurements cannot distinguish among plant uptake, microbial immobilization, leaching, precipitation, or redistribution into less soluble pools. Similarly, the concurrent lower OM and CEC values are consistent with the contribution of organic matter to exchange capacity, particularly in soils where organic functional groups provide important nutrient-retention sites (Oorts et al., 2003; Soares and Alleoni, 2008). In contrast, repeated long-term organic matter inputs can increase OM and CEC (Lee et al., 2023; Mäder et al., 2002; Ramos et al., 2018), indicating that the pattern observed here should not be generalized as an expected response to microbial treatments. The lower total N and NH₄^+^–N concentrations, together with unchanged NO₃^−^–N, do not support a simple interpretation of enhanced nitrification. These patterns may instead reflect combined effects of altered mineralization, microbial immobilization, plant uptake, volatilization, or redistribution among soil N pools (Gurmesa et al., 2022; Kuzyakov and Xu, 2013; Wang et al., 2022). In contrast, the increase in available P is consistent with microbial P mobilization through mineralization of organic P, phosphate solubilization, organic acid production, phosphatase activity, or competition for phosphate-sorption sites (Alori et al., 2017; Gyaneshwar et al., 2002; Menezes-Blackburn et al., 2018; Pang et al., 2024; Sharma et al., 2013). Because soil pH remained stable, the higher extractable P was unlikely to be attributable to a substantial bulk-soil pH shift. Nevertheless, elevated available P does not directly demonstrate greater plant P uptake or P-use efficiency because plant tissue nutrient concentrations and individual soil P fractions were not measured.

The correlation analysis further indicated coordinated variation among several soil chemical properties. EC, CEC, OM, total N, NH₄^+^–N, and exchangeable K, Ca, and Mg showed strong positive covariation, whereas available P generally varied in the opposite direction. This pattern is consistent with the retention of NH₄^+^ and exchangeable base cations on organic matter and soil exchange sites, while NO₃^−^ is comparatively mobile and showed weak relationships with most other soil chemical variables (Bel et al., 2020; Geisseler and Scow, 2014; Gurmesa et al., 2022; Nannipieri et al., 2017). The limited relationship between pH and the other variables may partly reflect the narrow pH range between NT and BT, despite the recognized importance of pH as a broad-scale driver of soil bacterial community structure (Rousk et al., 2010; Tripathi et al., 2018). Importantly, however, these correlations were calculated across only six samples by pooling the NT and BT observations without statistically separating treatment effects. Consequently, some of the high correlation coefficients likely reflect the overall chemical separation between the two treatments rather than intrinsic relationships among individual soil properties. The correlation matrix should therefore be interpreted as exploratory treatment-associated covariation rather than as evidence of direct functional relationships among soil variables.

Rarefaction analysis indicated that all six samples could be compared at a common sequencing depth, although the curves did not reach complete asymptotes and therefore do not demonstrate exhaustive recovery of bacterial richness. Bray–Curtis dissimilarity showed an apparent treatment-associated clustering pattern between NT and BT; however, this separation was not statistically supported by PERMANOVA (pseudo-*F* = 1.464, *R*^2^ = 0.268, *p* = 0.100). Thus, the present dataset does not provide statistical evidence for a treatment-driven difference in overall bacterial community composition, despite the descriptive clustering observed among samples. Proteobacteria and Acidobacteria remained dominant in both treatments, consistent with their common occurrence in blueberry-associated rhizospheres (Jia et al., 2024; Yurgel et al., 2017; Zhang et al., 2021). Several other phyla showed differences in mean relative abundance between NT and BT, but these patterns should be regarded as descriptive because phylum-specific statistical differences were not independently established. Previous studies have shown that blueberry rhizosphere bacterial composition can vary with cultivation conditions, soil properties, plant developmental status, and microbial inoculation, emphasizing the context dependence of apparent community shifts (Che et al., 2022; Wang and Yang, 2024). The higher observed richness, Shannon diversity, and Simpson diversity in BT was restricted to the treatment-responsive bacterial sequence-feature subset and therefore should not be interpreted as evidence that total-community alpha diversity was greater in BT. Instead, these results indicate greater diversity within the selected treatment-responsive feature set, consistent with the broader observation that rhizosphere responses to microbial inputs depend on inoculant identity, soil conditions, nutrient status, and treatment duration (Papin et al., 2024; Shen et al., 2021).

The CCA and Mantel analyses further indicated covariation between selected bacterial groups and the contrasting soil chemical environments of NT and BT. In the revised CCA, CCA1 and CCA2 accounted for 61.42 and 30.67% of the constrained variation, respectively. Available P was oriented toward negative CCA1, whereas CEC and NH₄^+^–N were oriented toward positive CCA1 and CCA2, broadly corresponding to the contrasting chemical characteristics observed between treatments. Exploratory Mantel screening identified nominal associations involving Acidobacteria, Actinobacteria, Bacteria_unclassified, Bacteroidetes, Firmicutes, Gemmatimonadetes, Nitrospirae, Planctomycetes, Proteobacteria, and Verrucomicrobia. Among the strongest nominal relationships were those involving Proteobacteria and A-P, Firmicutes and Ca, and Nitrospirae with K and NH₄^+^–N. However, Mantel r represents correspondence between distance matrices and does not indicate that the abundance of a taxon increased directly with the measured soil property. Moreover, because multiple Mantel tests were performed without multiple-testing correction, these relationships should be treated as exploratory rather than as confirmed taxon–environment associations. This cautious interpretation is consistent with evidence that edaphic variation can contribute strongly to the organization of blueberry rhizosphere communities without establishing direct causal effects of individual soil variables on particular bacterial taxa (Zhang et al., 2017; Zhao et al., 2019).

At the genus level, 68 genera or unclassified taxonomic groups showed nominal treatment-associated abundance patterns. BT showed higher representation of multiple taxa within Acidobacteria, Bacteroidetes, Firmicutes, Gemmatimonadetes, Proteobacteria, and Verrucomicrobia, whereas other taxa within the same phyla were more abundant in NT. For example, Bacillus, Gaiella, Bradyrhizobium, Roseisolibacter, and several unclassified taxa were more strongly represented in BT, whereas Terrimonas, Rhodomicrobium, Rhodoplanes, Nitrosomonadales_unclassified, and several other Proteobacteria-related taxa were more strongly represented in NT. The occurrence of Bacillus and Bradyrhizobium is consistent with previous observations that these taxa can occur as important members of blueberry-associated bacterial communities (Jia et al., 2024). However, the large positive or negative log₂ fold-change values for taxa absent or nearly absent in one treatment are strongly influenced by zero or near-zero abundances and should therefore be interpreted mainly as treatment-associated occurrence patterns rather than direct estimates of population growth or decline. The higher relative abundance of Bradyrhizobium in BT does not demonstrate biological N fixation because blueberry is not a leguminous host and the present 16S rRNA dataset did not identify nitrogen-fixation genes or strain-level functional traits. Likewise, the lower representation of Nitrosomonadales_unclassified and the descriptive treatment difference in Nitrospirae are compatible with altered N-related microbial composition but do not demonstrate changes in ammonia oxidation or nitrification without measurements of nitrification rates or functional genes such as amoA (Cai et al., 2021; Jones et al., 2016; Prosser and Nicol, 2012; Tourna et al., 2011). More generally, because the genus-level *p*-values were nominal and were not corrected for multiple comparisons, these taxonomic patterns should be interpreted as exploratory rather than as statistically confirmed differential abundance.

Predicted functional profiles also differed between NT and BT within the treatment-responsive bacterial sequence-feature subset. At the nominal *p* < 0.05 threshold, the displayed pathways with higher predicted abundance in BT included functions related to amino acid biosynthesis and metabolism, nucleotide metabolism and salvage, carbohydrate and central carbon metabolism, cofactor biosynthesis and salvage, cell-envelope-associated metabolism, and nutrient-related pathways such as assimilatory nitrate reduction. In contrast, only four displayed pathways showed lower predicted abundance in BT, including sucrose degradation II, the superpathway of (Kdo)₂-lipid A biosynthesis, and two chlorophyllide a biosynthesis pathways. These functional predictions should not be interpreted as an independent confirmation of the taxonomic results because PICRUSt2 derives functional potential from phylogenetic placement of 16S rRNA sequence features and reference genomes (Douglas et al., 2020). Moreover, the pathway comparisons were based on nominal *p*-values without multiple-testing correction and were generated from a selected treatment-responsive sequence-feature subset rather than the entire bacterial community. Therefore, the results represent exploratory predictions of functional potential and do not demonstrate differences in gene abundance, transcription, enzyme activity, or metabolic flux (Douglas et al., 2020; Matchado et al., 2024). This limitation is particularly important for PWY-7111, annotated as “pyruvate fermentation to isobutanol (engineered),” which should be viewed as a database-derived functional annotation rather than evidence that isobutanol fermentation occurred in the blueberry rhizosphere (Carmona et al., 2009; Caspi et al., 2020; Karp et al., 2019). Taken together, earlier fruit set and higher SSC in BT occurred concurrently with higher available P, differences in selected bacterial taxa, and altered predicted functional profiles. However, rhizosphere chemistry and bacterial communities were assessed only at the end of the cultivation period, so the present study cannot establish whether these rhizosphere differences contributed to the fruit responses, resulted from differences in plant development, or developed concurrently. Furthermore, because no separate sterile culture-medium control was included, the BT–NT contrast cannot distinguish effects attributable specifically to viable microbial cells from potential effects of components introduced with the culture media. The overall findings should therefore be interpreted as coordinated treatment-associated responses rather than as evidence of a demonstrated causal pathway from microbial inoculation through rhizosphere modification to fruit development.

## 5 Conclusion

Microbial treatment was associated with earlier fruit set and higher soluble solids content in blueberry, together with marked differences in rhizosphere soil chemical properties, particularly lower EC, CEC, nitrogen-related parameters, exchangeable cations, and organic matter, and higher available phosphorus. Although bacterial communities showed an apparent treatment-associated clustering pattern, this separation was not statistically supported by PERMANOVA. Nevertheless, treatment-responsive bacterial sequence features showed distinct taxonomic patterns and exploratory associations with soil chemical properties.

PICRUSt2 further suggested treatment-associated differences in predicted bacterial functional potential, particularly in pathways related to amino acid, nucleotide, carbon, cofactor, and nutrient metabolism. These results should be interpreted as exploratory because the functional profiles were inferred from 16S rRNA gene data and multiple pathway comparisons were not corrected for multiple testing.

Overall, the findings indicate that microbial treatment was associated with coordinated changes in blueberry fruit traits, rhizosphere nutrient conditions, and bacterial community characteristics. However, the present study does not establish a causal relationship among these responses, and the effects of viable microorganisms could not be separated from those of culture-medium components. Further replicated studies incorporating temporal sampling and direct functional measurements are needed to clarify the underlying mechanisms.

## Data Availability

The datasets presented in this study can be found in online repositories. The sequencing data generated in this study have been deposited in the National Center for Biotechnology Information (NCBI) Sequence Read Archive (SRA) under BioProject accession number PRJNA1524128.
